# Defining epitope coverage requirements for T cell-based HIV vaccines: Theoretical considerations and practical applications

**DOI:** 10.1186/1479-5876-9-212

**Published:** 2011-12-08

**Authors:** Jeffrey R Currier, Merlin L Robb, Nelson L Michael, Mary A Marovich

**Affiliations:** 1 US Military HIV Research Program (MHRP), Rockville, MD, USA

## Abstract

**Background:**

HIV vaccine development must address the genetic diversity and plasticity of the virus that permits the presentation of diverse genetic forms to the immune system and subsequent escape from immune pressure. Assessment of potential HIV strain coverage by candidate T cell-based vaccines (whether natural sequence or computationally optimized products) is now a critical component in interpreting candidate vaccine suitability.

**Methods:**

We have utilized an N-mer identity algorithm to represent T cell epitopes and explore potential coverage of the global HIV pandemic using natural sequences derived from candidate HIV vaccines. Breadth (the number of T cell epitopes generated) and depth (the variant coverage within a T cell epitope) analyses have been incorporated into the model to explore vaccine coverage requirements in terms of the number of discrete T cell epitopes generated.

**Results:**

We show that when multiple epitope generation by a vaccine product is considered a far more nuanced appraisal of the potential HIV strain coverage of the vaccine product emerges. By considering epitope breadth and depth several important observations were made: (1) epitope breadth requirements to reach particular levels of vaccine coverage, even for natural sequence-based vaccine products is not necessarily an intractable problem for the immune system; (2) increasing the valency (number of T cell epitope variants present) of vaccine products dramatically decreases the epitope requirements to reach particular coverage levels for any epidemic; (3) considering multiple-hit models (more than one exact epitope match with an incoming HIV strain) places a significantly higher requirement upon epitope breadth in order to reach a given level of coverage, to the point where low valency natural sequence based products would not practically be able to generate sufficient epitopes.

**Conclusions:**

When HIV vaccine sequences are compared against datasets of potential incoming viruses important metrics such as the minimum epitope count required to reach a desired level of coverage can be easily calculated. We propose that such analyses can be applied early in the planning stages and during the execution phase of a vaccine trial to explore theoretical and empirical suitability of a vaccine product to a particular epidemic setting.

## Background

Despite advances in therapeutic and prevention campaigns an estimated 33.4 million people currently live with HIV/AIDS and in 2008 alone, an estimated 2.7 million new infections occurred [1]. It is therefore generally accepted that controlling the global HIV pandemic will require a successful HIV/AIDS vaccine [2-5]. The design of an effective HIV vaccine is a multifaceted challenge involving the development of novel vectors, adjuvants and antigen design and formulation strategies. The elicitation of HIV-specific CD4^+ ^and/or CD8^+ ^T cells is considered an essential component of any potentially efficacious vaccination strategy [4,6-8]. Such cells could act as direct effectors in limiting virus replication, or as helper cells facilitating other effector mechanisms [9-13]. While a comprehensive review of the evidence supporting a T cell based HIV vaccine is beyond the scope of this introduction, a T cell based vaccine should preferably elicit an immune response capable of preventing infection or, at a minimum, result in control of viral replication after infection [14-16]. The recent Thai phase III trial (RV144) remains an important example of a vaccine that may require appropriate T cell help its protective effect through adequate antibody generation [17].

T cells recognize virally infected target cells or antigen-presenting cells via T cell receptor binding to short peptides (typically 8-12 amino acids in length) presented in the context of HLA class I or class II alleles [18-21]. It is the exquisite specificity of this interaction that determines activation and subsequent function of the T cell [22-24]. Since even a single conservative amino acid substitution within an epitope may alter or ablate appropriate T cell function, it becomes readily apparent why viral genetic diversity is foremost of the many daunting obstacles that have impeded the successful development of an HIV vaccine. The ability of HIV-1 to mutate and rapidly evolve to evade host immune pressure is mediated by an error-prone reverse transcriptase, genetic recombination and the host-derived APOBEC editing enzymes [25-28]. Due to the vast diversity of potential challenge viruses that constitute the global HIV-1 pandemic much effort recently has been focused on designing vaccine insert sequences to reduce the impact of viral diversity. These approaches include, but are not limited to, synthetic constructs that result in conserved minimal epitope (polyepitope) and conserved element concatenation into pseudoprotein strings, computationally derived constructs such as consensus, ancestral, center-of-tree and mosaic sequences, as well as cocktails of natural sequences derived directly from HIV-1 [26,29-41]. Regardless of which of these insert design strategies ultimately proves to be the most successful, the critical question of how best to assess and impartially compare these products in pre-clinical studies or the setting of a vaccine trial remains open. While several reviews, studies and experimental approaches have focused on how to deal with the question of viral diversity in the context of immunogen design, to date no study has focused upon how diversity coverage may be meaningfully assessed in the setting of an HIV vaccine trial [26,32,34,39,42].

Mounting data suggests that the breadth (number of discrete epitopes) and depth (amount of epitope variants recognized) of the T cell response is an important determinant of both HIV pathogenesis and primate model SIV vaccine efficacy. Multiple studies have recently indicated that it is the breadth of the T cell response rather than the magnitude, which correlates best with viral load set point in chronic HIV infection [43-50]. In addition a seminal study in the rhesus macaque SIV model demonstrated that immune control of an SIV challenge by a T cell-based vaccine was correlated with the breadth of vaccine generated T cell responses [51]. The fundamental premise of this study was to determine the extent of coverage a particular vaccine product would provide for either the global HIV pandemic or for the homologous subtype from which the product was derived, and to assess the relationship between epitope breadth and depth, and vaccine coverage. In this study, it is shown that by incorporating epitope breadth and depth analysis into vaccine coverage assessment, metrics can be used to determine the suitability of a particular vaccine formulation for a given setting. Parameters such as the level of coverage of possible incoming HIV-1 strains (75%, 90% or 95%), the sample set of possible strains (single subtype versus global group M) and the number of desired exact epitopes matches with an incoming strain (1-, 2-, or 3-hit models) are all shown to place dramatically different requirements upon epitope breadth. Furthermore, increasing the valency (number of included HIV-derived inset sequences) of the vaccine formulation is shown to dramatically increase the breadth of coverage and that for even natural sequence-based products, coping with the enormous genetic diversity of HIV may not be a totally intractable problem. While this study has focused upon extant natural sequence-based vaccine products that are currently under development, the analysis presented could easily be performed on new generation computationally derived vaccine products. Not only does this approach provide an empirical assessment of vaccine coverage potential based upon the formulation, but it also allows direct theoretical comparison of vaccine products as they might apply to a particular epidemic of HIV-1. Finally, using example data from a vaccine trial it is shown that the theoretical calculations compare well with the experimentally generated data.

## Methods

### Query sequences and test datasets

The source of all natural HIV-derived Gag and Env protein query sequences used in the study were a series of recombinant modified vaccinia Ankara (rMVA) constructs that have been developed at the Military HIV Research Program (MHRP) [31]. Each product contains a native *gag*/*pol *open reading frame and a native *env *open reading frame with a C-terminal truncation of gp41 to maximize expression. The products are based upon isolates representing subtype A (MVA-KEA), subtype C (MVA-TZC), subtype D (MVA-UGD), and CRF01_AE (MVA-CMDR). Additional File 1 describes each product in detail together with the isolate sequence of origin and GenBank accession number. Test sequence datasets were obtained from the Los Alamos National Laboratory HIV Sequence Database http://www.hiv.lanl.gov/content/sequence/HIV/mainpage.html using the 2009 alignments for HIV-1. The final sequence sets were filtered to remove all N- and O-group and Cpz origin viruses and retain all M-group sequences to form a dataset without bias for subtype or recombinant form inclusion. The final Gag and Env query datasets consisted of 3585 and 2258 sequences respectively.

### Calculation of T cell-based vaccine coverage potential

To quantify the coverage that a particular HIV vaccine insert may provide in terms of T cell recognition an N-mer identity approach was used to scan for epitope matches between vaccine product test sequences and a query dataset of global M-Group and subtype-specific HIV Gag and Env sequences. For the purposes of this study epitopes are defined as 10 amino acids in length since this represents a reasonable compromise between the median CD8^+ ^T cell epitope length (9-10 amino acids) and the minimal core of a CD4^+ ^T cell epitope (10-12 amino acids). The Epicover algorithm (epitope coverage assessment tool; [32,52]) as implemented in the LANL HIV Sequence Database http://www.hiv.lanl.gov/content/sequence/MOSAIC/epicover.html was used to calculate the proportion of HIV Gag or Env isolate sequences from the query datasets above which have an exact 10-mer match with a given vaccine sequence, or cocktail of sequences. Analysis of exact matches occurs by dividing the query sequence(s) into all possible 10-mers (the vaccine "epitome"), then walking each 10-mer along the test sequences in one amino acid "windows" and scoring each test sequence for a hit (1), or no hit (0). The coverage potential of the vaccine is then expressed as a percentage (or fraction) of sequences in the test dataset for which an exact 10-mer match is made. Epicover calculates and expresses coverage as the mean coverage of N-mers from all test sequences and is not a strict "hit/no-hit" model of N-mer matching for each individual sequence. As shown in Additional File 2, Epicover produces virtually identical results to a strict "hi/no-hit" algorithm when a sufficiently large dataset is used and the query sequence dataset (vaccine cocktail) produces a near Gaussian distribution of random N-mer matches with the test sequence dataset.

### Calculation of epitope requirements for vaccine coverage

The above calculation of vaccine coverage assumes that each vaccine is capable of generating only a single T cell epitope, which in both theoretical and practical terms is not the case. Incorporation breadth (number of discrete epitopes) and depth (amount of epitope variants recognized) of epitope recognition into the vaccine coverage analysis is performed at two stages. Depth was included in the epitope coverage analysis above by considering multivalent vaccines and each of the different epitope variants they might present. Analysis of breadth was included by considering the total number of epitopes that may be generated by a given vaccine modality. To integrate the capacity of vaccines to generate multiple epitopes into the analysis, the following calculation was used:

p(n)=p(n-1)+p(n-1)×(1-p(n-1)

Where: *n *is the number of epitopes generated by a vaccine

*p *is the probability of an exact hit on the query dataset

This calculation can be iterated for as many epitopes as considered practically possible for a vaccine. The initial *p*(*n*-1) value for the formula is derived from the Epicover calculation for single epitope coverage. The resulting coverage potential of a vaccine should therefore increase as the breadth and depth of the T cell response increases. Positional coverage analysis was performed by aligning the LANL 2009 Gag dataset to the HXB2 reference sequence and performing gap stripping where necessary to maintain the consensus sequence. Coverage provided at each of 493 10-mer windows across the 502 amino acid Gag HXB2 was plotted on a frequency histogram and examples "low" and "high" depth of coverage epitopes were annotated.

### Calculation of epitope requirements for vaccine coverage using "multiple hit requirement" models

The prior analyses all work on the implicit assumption that a single exact epitope match (or "hit") on a potential incoming strain of HIV-1 will be sufficient for vaccine efficacy. This assumption may be flawed if we consider that more than one exact match with a potential incoming strain of virus may be required. Such a requirement can be thought of in terms of a "multiple hit" model where two or more exact epitope matches are required with any incoming strain of virus for killing of the infected cell or inhibition of virus replication and preventing viral escape. This model is best described using the exact binomial probability calculation:

$$p\left(k\;\text{out}\;\text{of}\;N\right)=\;\frac{N!}{k!\left(N-K\right)!}\left(p^{k}\right)\left(q^{N-k}\right)$$

Where: *k *= the number of exact epitope matches required (or hits)

*N *= the actual number of epitopes generated by a vaccine

*p *= probability that a single exact epitope match occurs

*q *= probability that a single exact epitope match does not occur

Applying this calculation to all values of *k *equal to or smaller than *N *and summing the values yields the probability of getting at least the required number of exact matches (hits) for any given epitope count. For example, when considering a 3-hit model, the probability of at least 3 exact matches with a vaccine that generates 10 epitopes would be calculated by:

$$p(\text{at}\;\text{least}\;\text{3}\;\text{hits}\;\text{out }\;\text{10})\;\text{ = }\overset{10}{\underset{k=3}{\sum }}\frac{N!}{k!\left(N-k\right)!}\left(p^{k}\right)\left(q^{N-k}\right)$$

For the purpose of simplicity the two-hit and three-hit models are considered for this study, however the models could be extended to four-hit or five-hits if empirical data supported this requirement.

### Application of breadth and depth analysis and multi-hit models of vaccine coverage to assessment of vaccine trial data

To demonstrate the application of breadth and depth analysis in the setting of a vaccine trial, a responder subject from a recently completed trial of the MVA-CMDR vaccine product (RV158; [53]) was assessed for breadth and depth of HIV-specific T cell responses. Briefly, 2 week *in vitro *stimulated PBMC cultures were established as previously described to generate effector cells for assessing HIV-specific cytotoxic activity [53]. The effector cells from a positive responder to both Gag and Env were then epitope mapped using a matrix of peptides matching the Gag and Env insert sequences of MVA-CMDR using and IFN-γ ELISPOT assay. Responses were de-convoluted and the identified epitopes enumerated and subjected to analysis for depth of coverage using the Epicover algorithm (epitope coverage assessment tool; [32,52]). The breadth of the response was used to determine coverage potential of homologous subtype (CRF01_AE) and the global group M epidemic as described above.

## Results

### Epitope breadth requirements for coverage of the global group M HIV-1 database with natural sequence constructs

**Figure 1:**
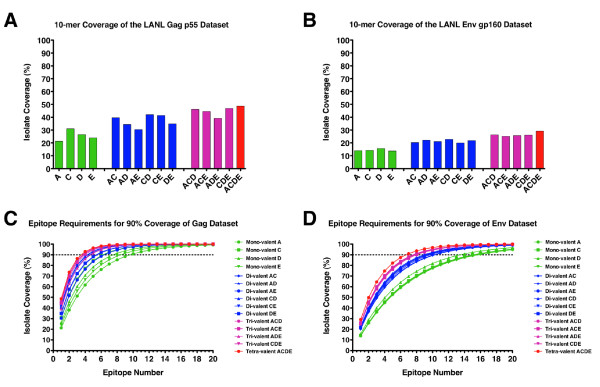
**Epitope coverage for four Gag and Env natural sequence products**. Epitope coverage analysis (exact 10-mer window matching) is shown for four different natural sequence Gag and Env protein-based vaccine products. Mean coverage of the global HIV-1 group M Gag (3585 sequences) and Env (2258 sequences) datasets obtained from the LANL HIV Database is shown as a percentage of the possible incoming isolates for which there would be an exact match at any 10-mer window (Panels **A **and **B**). Green bars represent the mono-valent Gag and Env sequences, while the blue, pink and red bars represent all possible di-valent, tri-valent and tetra-valent formations of the four individual products respectively. Based upon this analysis, the coverage that each formulation would provide was calculated and plotted for up to 20 T cell epitopes (Panels **C **and **D**). Color coding of the lines matches panels A and B above (green for mono-valent, blue for di-valent, pink for tri-valent and red for tetra-valent). The point at which each formulation achieves 90% coverage of all isolates in the LANL database is denoted by the dotted line.

Initially the global HIV-1 group M coverage that would be provided by the Gag and Env natural sequence inserts in the MHRP rMVA product development pipeline was determined (tabulated in Additional File 1). As stated in the Methods section 10-mer amino acid windows were defined as the epitope length unit for this study. The Epicover algorithm (LANL HIV Database) was then used to determine the theoretical coverage provided a single epitope generated by the Gag or Env sequences in each vaccine product for the global M-group pandemic (3585 Gag sequences; 2258 Env sequences). The exact 10-mer epitope coverage provided by the four natural Gag sequences ranged from 21.3% to 31.0%, while the coverage provided by the four Env sequences ranged from 13.7% - 15.7% (Figure 1A and 1B; green bars). This is in agreement with the published data of Korber et al [26] for analysis of individual natural sequences for coverage of the LANL HIV Database datasets. Subsequently, the coverage provided by all possible multivalent combinations (di-, tri- and tetravalent) was calculated for the 4 Gag and Env natural sequences. As would be predicted the coverage potential of the multivalent cocktails increases as the valency increases due to the unique diversity of each individual construct. The coverage range increases to 30.4% - 42.0% for the divalent Gag cocktails (Figure 1A, blue bars), to 39.2% - 46.9% for the trivalent Gag cocktails (Figure 1A, pink bars), while the tetravalent Gag cocktails present the greatest coverage potential of 48.7% (Figure 1A, red bar). Similar increases in coverage potential were seen for the Env natural sequence cocktails (Figure 1B). Due to the remarkable diversity of group M Env proteins, coverage potential is limited to 29.2% for the tetravalent Env cocktail.

**Figure 2:**
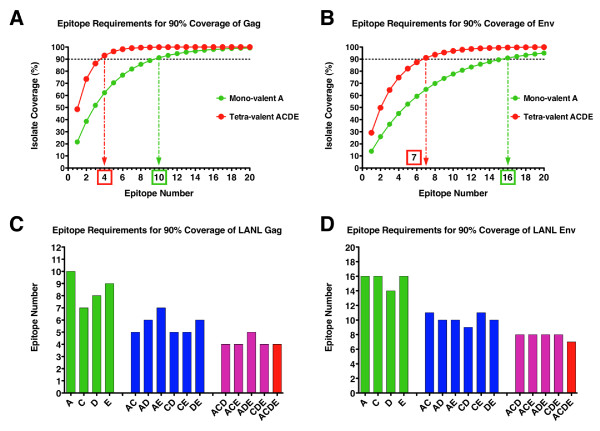
**Calculation of minimum epitope coverage requirements**. Interpolation of the required minimum epitope count for achieving 90% isolate coverage for mono-valent subtype A and tetra-valent vaccine formulations (ACDE) is shown for Gag (Panel **A**) and Env (Panel **B**). Calculated minimum epitope counts required to achieve 90% coverage all isolates in the LANL database is shown as a bar graph for Gag (Panel **C**) and Env (Panel **D**). Color coding of the bars matches Figure 1 above (green for mono-valent, blue for di-valent, pink for tri-valent and red for tetra-valent).

This analysis assumes that a single epitope is generated by the vaccine construct/cocktail in question. Increasing the breadth or total number of epitopes generated by a given vaccine construct amplifies the coverage potential dramatically. Figures 1C and 1D show the theoretical coverage potential of all mono-, di-, tri- and tetravalent formulations for the Gag and Env natural sequences respectively. Coverage is shown for the vaccines generating a range of 1 through 20 discrete T cell epitopes. This represents the boundaries of a practical range of possible discrete T cell epitopes that a vaccine might be able to generate. It can be seen that a point of diminishing return is eventually reached for each curve as it becomes asymptotic with the theoretical limit. Using such an analysis it is possible to set a limit of minimal coverage that would be required for a vaccine product to achieve. In the case shown here (and used throughout this analysis) a coverage limit of 90% has been applied. By interpolating along the coverage requirement for a vaccine, the epitope count that is required to meet or exceed this requirement can be calculated. Figures 2A and 2B show examples of interpolating the minimal epitope count required to meet 90% coverage of any incoming M-group isolate of HIV-1 for the Gag and Env natural isolates respectively. The examples shown here highlight the clear advantage that a multi-valent cocktail provides when compared with a mono-valent product. When considering Gag-specific responses the mono-valent subtype A natural sequence would need to generate a median of 10 epitopes per subject to reach 90% coverage of the global M-group dataset, whereas the tetra-valent cocktail would achieve the same coverage by generating a median of 4 epitopes per subject (Figure 2A). Due to greater envelope sequence diversity the epitope requirements to reach 90% coverage are increased to 7 epitopes for the tetra-valent cocktail and 16 epitopes for the mono-valent subtype A natural sequence (Figure 2B). As predicted by the coverage potential calculations, the epitope requirements to reach 90% coverage of the global M-group dataset decreases as the vaccine valency increases for both Gag and Env respectively (Figures 2C and 2D). The multi-valent cocktails provide better coverage with fewer epitopes due to the presence of different epitope variants in the individual isolate sequences. The increase in depth of coverage of any given epitope derives from a greater number of epitope variants contained in higher valency formulations. This means that for a given vaccine sequence and valency and a given dataset representing the diversity of potential incoming viruses, a median epitope count threshold can be set as an important metric for measuring and comparing candidate HIV vaccines.

### Consideration of multiple hit requirements for T cell-based HIV vaccines

**Figure 3:**
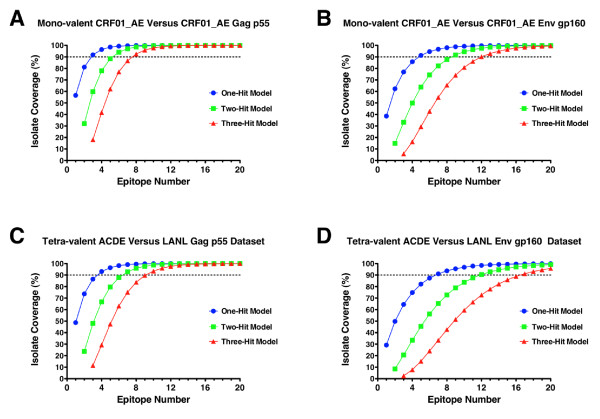
**Comparison of epitope requirements for vaccine coverage in multiple-epitope hit models**. Comparisons of 1-, 2- and 3-exact hit epitope requirement models are shown for a mono-valent product (MVA-CMDR Gag and Env inserts) assessed against a dataset of homologous subtype (CRF01_AE) derived sequences (88 Gag sequences, Panel **A**; 112 Env sequences, Panel **B**). For contrast a tetra-valent formulation of the products (ACDE) assessed against the entire HIV-1 group M Gag (Panel **C**) and group M Env (Panel **D**) datasets is also shown. In all graphs the 1-hit model is represented by the blue line and symbols, the 2-hit model is the green line and symbols and the 3-hit model is the red line and symbols. For reference, the 90% coverage point for each dataset is again denoted by a dotted line on each graph.

The prior analysis is predicated on the notion that a single T cell epitope match with an incoming strain of HIV would be sufficient for recognition of an infected target cell and prevention of productive infection. While conceptually valid this assumption may be an over simplification of the complexities of antigen processing and presentation and the differential kinetics of viral protein expression, viral epitope entropy and the TCR repertoire. Hence, some epitopes may provide more effective protection or control of HIV infection than others and so the chances of eliciting a response against a "good" epitope increases as the breadth of the response increases. To incorporate this potential constraint into the assessment of epitope requirements for T cell-based vaccines, a multiple hit model was incorporated into the analysis. Using such a model, two or more exact epitope matches would be required with any incoming strain of virus for vaccine efficacy. As described in the Methods section this model is a generalized "*k*" out of "*N*" type problem and can be described by exact binomial probability calculations. To illustrate how the requirement for multiple exact epitope hits impacts the overall epitope breadth requirement for vaccine coverage, the mono-valent CRF01_AE natural sequence (MVA-CMDR Gag and Env inserts) were first assessed for coverage of the homologous subtype (88 Gag sequences and 112 Env sequences from the 2010 LANL database). As shown in Figure 3 (panels A and B), the coverage of both Gag and Env are appreciably impacted as the exact hit requirement increases. In the example shown, the number of epitopes required to reach 90% coverage of the CRF01_AE Gag dataset increases from 3 to 6 to 8 epitopes as 1-, 2- and 3-hit models are considered respectively. For 90% coverage of the CRF01_AE Env dataset the epitope requirements increase even more dramatically from 5 to 9 to 12 epitopes for the 1-, 2- and 3-hit models respectively. When a tetra-valent formulation of the four vaccine products is considered for coverage of the global Gag and Env datasets (3585 Gag sequences; 2258 Env sequences) the impact of the multiple hit models on epitope requirements is quite stark (Figures 3C and 3D). In the case of the 3-hit model 90% coverage of the Gag and Env datasets require 10 and 17 epitopes respectively.

### Comparative analysis of different vaccine formulations and multiple-hit models of vaccine requirements

**Figure 4:**
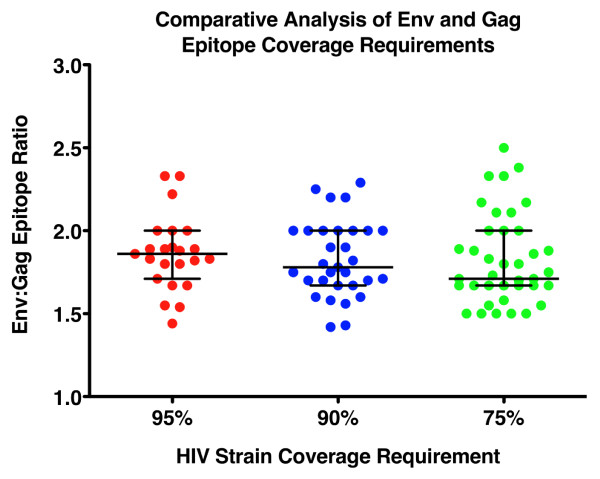
**Comparison of Env and Gag epitope coverage requirements**. Shown is the ratio of Env:Gag epitope requirements to reach 95%, 90% or 75% coverage of the global HIV-1 group M datasets for any given formulation of the four natural sequence-based products. Regardless of the valency of the vaccine product Env consistently requires approximately two-fold more epitopes to be generated than Gag to reach any given level of coverage.

Analysis of epitope requirements and incorporation of multiple hit requirements demonstrates that it is possible to set standards for HIV vaccine trials in terms of the mean number of epitopes generated per vaccinated subject as a metric of potential vaccine coverage in different settings of HIV diversity and for different vaccine insert formulations. Comparative analyses considering all possible mono-, di-, tri- and tetra-valent formulations for all four MHRP vaccine products considered in this study were performed and are included in Additional File 3 and Additional File 4. Notably it is possible to compare the relative benefits that multi-valent formulations may provide for increasing the breadth of vaccine coverage and decreasing the epitope requirements for maximizing global (or localized) vaccine coverage. Tables 1 and 2 summarize the epitope requirements for 95%, 90% and 75% coverage of the global group M Gag and Env datasets respectively. The epitope requirements for 95% and 75% coverage of any possible incoming strain of HIV-1 have also been included for comparison purposes as these may represent necessary, or preferred, alternative goals to the 90% coverage limit that has been applied throughout the prior analyses. Several important observations can be made from this analysis. First, it is clear that there is a large difference in the individual coverage provided by the 4 different natural sequence products (mono-valent analyses). This is particularly evident among the Gag sequences where the subtype C based construct (MVA-TZC) gives the best coverage and is most likely due a slight overrepresentation of subtype C derived sequences in the dataset relative to the subtypes from which the other three natural sequences were derived. Secondly, vaccines generating T cells that target the Gag protein may be advantageous in comparision to Env protein targeting vaccines. For any vaccine formulation valency and any exact hit requirement model considered there are approximately two-fold more Env epitopes required to reach any coverage requirement than for Gag (Tables 1 and 2). The impact of Env sequence diversity on epitope requirements is evident as medians of 1.9, 1.8 and 1.7 fold greater epitope requirements than Gag for coverage levels of 95%, 90% or 75%, respectively (Figure 4). However, it should be noted that the inclusion of Env in the vaccine constructs considered was also for the purpose of generating antibodies against native HIV sequences. The multi-valent formulations clearly provide a potential solution to the genetic "diversity problem" posed by HIV. For Gag, and even when considering a multi-hit model, the number of epitopes required to reach 90% coverage of the global group M Gag sequence database is 10 epitopes for the tetravalent formulation and for each of the 4 possible tri-valent formulations is 10 to 12 epitopes. While this is still a considerable number of epitopes it is not beyond the capacity of the immune system to generate such broad responses in the context of HIV-1 natural infection [47,54]. The Env protein again demonstrates the problem of genetic diversity that HIV-1 poses. Even for multi-valent Env formulations the number of epitopes required to achieve 90% coverage of the global group M Env sequence database ranges from 7 to 8 epitopes for the 1-hit model and rises to 17 to 20 epitopes for the 3-hit model. Generating this breadth of response presents a possibly intractable problem as it may be beyond the capacity of the immune system to generate such a high epitope number against a single protein. This result however should not be interpreted as a reason to preclude the inclusion of Env proteins in T cell based vaccine modalities. The data shown in Figure 3B clearly demonstrates that an appropriately selected, subtype matched, natural Env sequence may provide sufficient coverage within the homologous subtype with as few as 5 epitopes. Therefore, counting the number of epitopes generated by different vaccines and factoring the diversity provided by the sequence of the vaccine inserts will be critical to assessing the coverage provided for a specific HIV epidemic.

**Table 1:** Gag epitope coverage requirements of all possible formulations of the four natural sequence based products for the global group M dataset.

Natural Sequence Construct	Minimum number of T Cell Epitopes Required to Reach Indicated Coverage of Group M Gag Dataset								
	
	1-Hit Model			2-Hit Model			3-Hit Model		
	
	95%	90%	75%	95%	90%	75%	95%	90%	75%
Mono-valent MVA-KEA (A)	13	10	6	20	17	12	> 20	> 20	18
Mono-valent MVA-TZC (C)	9	7	4	14	12	8	19	16	12
Mono-valent MVA-UGD (D)	10	8	5	16	14	10	> 20	19	14
Mono-valent MVA-CMDR (E)	12	9	6	19	15	11	> 20	> 20	16
Di-valent KEA/TZC (AC)	6	5	3	10	9	6	14	12	9
Di-valent KEA/UGD (AD)	7	6	4	12	10	7	16	14	11
Di-valent KEA/CMDR (AE)	9	7	4	14	12	8	19	16	12
Di-valent TZC/UGD (CD)	6	5	3	10	8	6	13	11	9
Di-valent TZC/CMDR (CE)	6	5	3	10	8	6	13	12	9
Di-valent UGD/CMDR (DE)	7	6	4	12	10	7	16	14	11
Tri-valent KEA/TZC/UGD (ACD)	5	4	3	9	7	5	12	10	8
Tri-valent KEA/TZC/CMDR (ACE)	6	4	3	9	8	6	12	11	8
Tri-valent KEA/UGD/CMDR (ADE)	6	5	3	11	9	6	14	12	9
Tri-valent TZC/UGD/CMDR (CDE)	5	4	3	9	7	5	12	10	8
Tetra-valent KEA/TZC/UGD/CMDR (ACDE)	5	4	3	8	7	5	11	10	7

**Table 2:** Env epitope coverage requirements of all possible formulations of the four natural sequence based products for the global group M dataset.

Natural Sequence Construct	Minimum number of T Cell Epitopes Required to Reach Indicated Coverage of Group M Env Dataset								
	
	1-Hit Model			2-Hit Model			3-Hit Model		
	
	95%	90%	75%	95%	90%	75%	95%	90%	75%
									
Mono-valent MVA-KEA (A)	20	16	10	> 20	> 20	19	> 20	> 20	> 20
Mono-valent MVA-TZC (C)	20	16	10	> 20	> 20	19	> 20	> 20	> 20
Mono-valent MVA-UGD (D)	18	14	9	> 20	> 20	17	> 20	> 20	> 20
Mono-valent MVA-CMDR (E)	> 20	16	10	> 20	> 20	19	> 20	> 20	> 20
Di-valent KEA/TZC (AC)	14	11	7	> 20	18	13	> 20	> 20	19
Di-valent KEA/UGD (AD)	12	10	6	20	17	12	> 20	> 20	17
Di-valent KEA/CMDR (AE)	13	10	6	> 20	17	12	> 20	> 20	18
Di-valent TZC/UGD (CD)	12	9	6	19	16	11	> 20	> 20	17
Di-valent TZC/CMDR (CE)	14	11	7	> 20	18	13	> 20	> 20	19
Di-valent UGD/CMDR (DE)	13	10	6	20	17	12	> 20	> 20	17
Tri-valent KEA/TZC/UGD (ACD)	10	8	5	17	14	10	> 20	19	14
Tri-valent KEA/TZC/CMDR (ACE)	11	8	5	17	14	10	> 20	20	15
Tri-valent KEA/UGD/CMDR (ADE)	11	8	5	17	14	10	> 20	19	15
Tri-valent TZC/UGD/CMDR (CDE)	10	8	5	17	14	10	> 20	19	15
Tetra-valent KEA/TZC/UGD/CMDR (ACDE)	9	7	5	15	12	9	20	17	13

### Further refinements of vaccine coverage analysis

**Figure 5:**
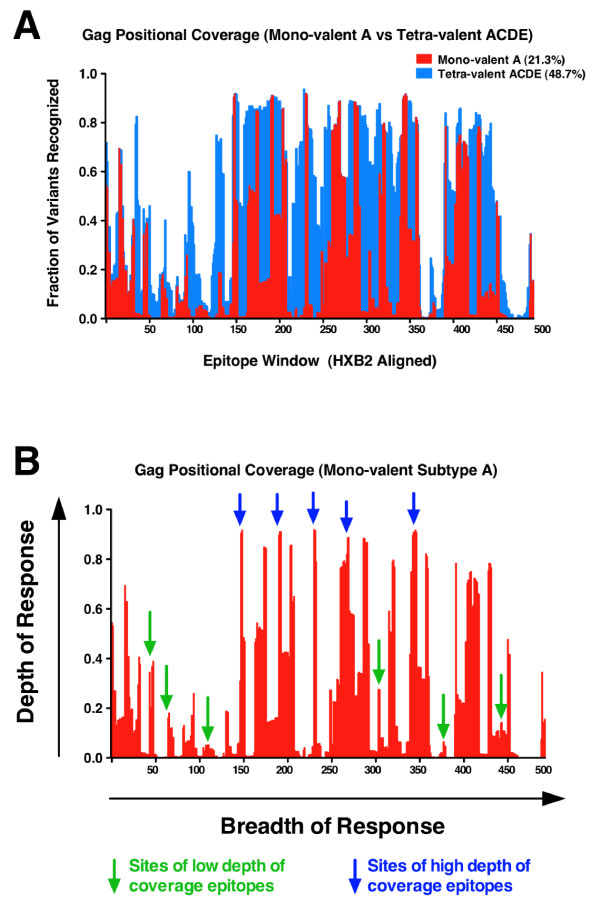
**Illustration of positional coverage for group M Gag sequences by a mono-valent versus a tetra-valent vaccine product**. Positional coverage of a group M Gag dataset (2385 sequences) is shown for the mono-valent subtype A vaccine product (MVA-KEA) and a tetra-valent formulation of the four products (ACDE). The fraction of variants with exact 10-mer matching in 493 successive windows of 1 amino acid across the Gag dataset (2385 proteins aligned to HXB2) for the monovalent formulation (red) and tetra-valent formulation (blue) is shown (Panel **A**). Potential examples of high- and low-depth of coverage epitopes are superimposed upon the positional coverage diagram to illustrate breadth and depth of coverage (Panel **B**).

While use of the mean 10-mer coverage that any given vaccine insert sequence provides is a practical solution to rapidly determining the theoretical epitope requirements, there are potential further refinements that can be made when considering vaccine coverage analysis. Figure 5A shows how the 10-mer coverage of any vaccine insert against a given query dataset, in this case the subtype A Gag protein coverage of the LANL group M Gag dataset, is a mean of the total positional coverage. The mono-valent subtype A coverage shown in red is a series of peaks and troughs representing regions of high and low depth of coverage across the group M Gag dataset. Also shown, superimposed in blue on the same figure, is the additional coverage provided by a tetra-valent ACDE vaccine formulation. The mean coverage for each vaccine product, mono-valent A (21.3%) and tetra-valent ACDE (48.7%), is the overall mean of an uneven discrete probability distribution spanning the entire protein. In Figure 5B examples are shown of how the exact position of an epitope can have a profound influence on the depth of coverage it can provide. Sites of high depth of coverage epitopes are shown in blue in contrast to sites of low depth of coverage epitopes shown in green. This example illustrates how the depth of a T cell epitope response is as important as knowing the breadth of the response. Two or three epitopes targeting highly conserved regions of a protein may be as effective as 8 to 10 epitopes targeting only sites of low coverage. Therefore not only counting the number of epitopes generated by a vaccine product, but also knowing their mapped positions adds further refinement to interpreting the potential coverage provided by a vaccine product.

As an example of how to practically assess the theoretical coverage that may be provided by a vaccine, data is presented from epitope mapping studies performed during a recently completed vaccine trial (RV158) involving one of the native sequence constructs (MVA-CMDR) considered in the prior analyses [53]. Epitope mapping was performed for Gag and Env from 2 different trial participants, the results of which are outlined in Additional File 5 and Additional File 6 for each participant respectively. As shown, one participant demonstrated a relatively broad response of 6 T cell epitopes in total (4 in Env and 2 in Gag), while the second subject demonstrated a narrow T cell response (2 epitopes) with a single epitope each in Env and Gag respectively. Using the methods described above and a 1-hit epitope requirement model the coverage provided by the response generated in each of these two participants is shown in Table 3. The effect of increased breadth upon HIV strain coverage is striking. The participant that generated 6 epitopes would have a 97.1% probability of making at least one exact epitope match with any incoming HIV strain from the homologous subtype (CRF01_AE) and a 67.7% probability of making an exact match with any global strain of HIV. In contrast, the participant with a single epitope against both Gag and Env would have 73.4% and 34.1% probability of making one exact epitope match within the homologous subtype or with a global strain of HIV respectively.

**Table 3:** Theoretical HIV isolate coverage provided by different vaccine trial responders.

		Subject 1			Subject 2	
	
	Gag	Env	Total	Gag	Env	Total
Number of Epitopes	2	4	6	1	1	2
Intra-subtype Coverage	81.1%	85.8%	97.3%	56.6%	38.6%	73.4%
(CRF01_AE)						
Global Group M Coverage	41.8%	44.5%	67.7%	23.7%	13.7%	34.1%

### Empirical assessment of T cell-based vaccine coverage

As a final refinement to the coverage analysis the sequence of the peptide(s) recognized from the vaccine product(s) can be used to directly query the database for depth of coverage independently. This represents both the most stringent form of coverage analysis and the most technically demanding and laboratory intensive analysis. Once the T cell epitopes have been enumerated, further studies can be performed to identify and confirm the peptides and/or peptide overlaps that are recognized. These short sequences can be used to screen databases and datasets for a more precise determination of the coverage provided. This affords a much greater precision for the coverage analysis calculations because as shown in Figure 5B, epitopes may fall on regions of high or low depth of coverage. Depending upon sample availability, the cross-reactive capacity of these responses with peptides representing other common variants utilized by HIV can provide a stringent picture of epitope depth of coverage. Empirical determination remains the only real means of assessing cross-reactivity since there currently no reliable computational methods which can gauge the cross-reactivity of T cells specific for a given epitope. Using the two examples used above (Additional File 5 and Additional File 6) the sequences of the peptides recognized were used to scan the global group M Gag and Env sequence datasets and the homologous subtype (CRF01_AE) for exact 10-mer matches within the 6 mapped epitopes (2 in Gag, and 4 in Env). Table 4 shows the exact 10-mer coverage provided by each of the 6 T cell epitopes identified in a vaccine trial participant. The Gag epitope VDRFYKTLRAE provides 89.7% and 42.1% coverage of the homologous subtype and global group M Gag epidemics respectively and would be considered a high depth of coverage epitope particularly for the CRF01_AE subtype. The second Gag epitope (NFPQSRPEPTAPPAE) also provides a medium level of coverage for subtype-specific and the overall group M Gag epidemics (58.6% and 42.4% respectively). This contrasts with the Env epitopes, which vary dramatically in their coverage of the homologous subtype and the overall group M Env datasets. Three of the Env epitopes would provide 45.1%, 30.6% and 20.7% coverage of the homologous subtype, putting them in the intermediate range of depth of coverage. However, the coverage provided by these epitopes for the group M global Env dataset would be only 2.8% to 6.9%, hence putting them in the low depth of coverage category for global Env diversity. The remaining Env epitope represents a unique sequence in the database and provides no exact 10-mer coverage. The overall potential coverage determined by both methods - enumeration of the epitopes (Table 3) and epitope mapping and sequence dataset screening (Table 4) - arrived at remarkably similar results. Overall coverage calculated by epitope enumeration for the homologous subtype and the global M-group was 97.3% and 67.7% respectively, which is comparable to the epitope mapping and screening method determinations of 98.7% and 71.1% for the corresponding datasets. This data therefore provides empirical validation of the epitope enumeration method as a rapid means of determination potential coverage in the setting of vaccine trial assessment. The enhancement that increased breadth of recognition provides is exemplified by the 69.9% overall coverage provided by the 4 Env epitopes corresponding to the homologous subtype. This implies that at least within a subtype, Env sequence diversity may not present an intractable problem for T cell-based vaccine development.

**Table 4:** HIV isolate coverage provided by identified epitope regions.

Protein	Epitope	Intra-subtype Coverage	Global Coverage
		(CRF01_AE)	(All Group M)
Gag	VDRFYKTLRAE	89.7%	42.1%
	NFPQSRPEPTAPPAE	58.6%	42.4%
	All Gag	95.7%	66.7%
			
Env	VHALFYKLDIVPIED	20.70%	6.9%
	EKLKEHFNNKTIIFQ	0%	0%
	EISNYTNQIYEILTE	45.10%	2.8%
	EPDRSERIEEGGGEQ	30.6%	5.7%
	All Env	69.9%	14.7%
			
Overall	Gag and Env	98.7%	71.6%

## Discussion

In this study it has been demonstrated that measurement and analysis of the breadth of the T cell response - the number of discrete T cell epitopes - generated by a vaccine provides an important metric for HIV vaccine assessment. Analysis of the breadth of T cell coverage not only illustrates the problem that HIV genetic diversity presents to the immune system, but it also demonstrates that increasing the breadth of coverage can amplify the capacity of even natural sequence-based vaccine products to cope with viral diversity. Practical metrics can be set prior to the commencement of a vaccine trial that take into account both the HIV sequence content in the vaccine product and the diversity of HIV in particular target settings. Importantly, the relative capacities of different multi-valent formulations to generate sufficient epitope coverage can be compared directly. While the analyses presented here are limited to exact 10-mer matches as epitope hits this was considered to be a reasonable compromise between the median CD8^+ ^T cell epitope length (9-10 amino acids) and the minimal core of a CD4^+ ^T cell epitope (10-12 amino acids). However, shorter or longer epitope windows (8, 9, 10, 11 or 12 amino acids) and more relaxed matching requirements (1 or 2 tolerated amino acid mismatches) could be incorporated as desired into this analysis. It was felt that the requirements used here represent a more conservative approach and hence may in fact over-estimate the epitope requirements since no mismatches are tolerated. The studies of Korber et al. [26], using computationally generated mosaic vaccine insert sequences clearly demonstrate a progression to increased vaccine coverage as matching requirements are relaxed and as smaller epitope windows are considered [26,32]. Importantly, the practical application of epitope mapping in vaccine evaluation demonstrates that understanding the depth of individual epitope coverage provides an added layer of sophistication to vaccine coverage assessment. Preclinical studies investigating the cellular immunogenicity of mosaic constructs and comparing them with natural sequence constructs unequivocally demonstrates the importance of breadth and analysis as a metric of the T cell response [55,56].

Empirical data has begun to emerge that implicates T cell epitope breadth as a critical parameter in both the natural control of HIV-1 infection and in pre-clinical testing of different vaccine strategies and modalities. In cross-sectional studies of naturally HIV-1 infected, treatment naïve human subjects, investigators have identified the breadth of the cellular immune response, rather than the magnitude of the response, as the primary discriminator of effective immune control as measured by set-point viral load [43,45,49,50,57,58]. Furthermore, the transmission of escape variants for Gag-specific CD8^+ ^T cell epitopes is associated with reduced viral load in linked subjects, and if multiple epitopes are targeted the likelihood of epitope reversion is diminished [49,50,59-61]. Computational analysis and epitope prediction based on HLA type and HIV proteome sequence in a large cohort of 302 subjects has also shown that epitope repertoire and breadth is critical to viral load control [48]. The seminal study by Liu and co-workers [51] demonstrated immune control of an SIV challenge by a T cell-based vaccine in rhesus macaques and linked breadth of vaccine generated T cell responses with relative efficacy of different immunogens. This study established for the first time a correlate of cellular immunity and mitigation of disease outcome in a realistic SIV challenge model. To date there is no clear indication of the exact breadth of the T cell response that the human immune system is capable of generating against the Gag and Env proteins considered here. However, pre-clinical data from the rhesus macaque model suggest that 20 or more T cell epitopes can readily be generated by vaccination with a non-replicating viral vector [55,56]. In natural HIV infection it has been shown that 20 or more epitopes can be detected within a single individual when the entire HIV proteome is considered [47,54]. Whether such breadth is practically attainable in the setting of a human vaccine study remains to be proven. In this study the generation of greater than 20 epitopes per protein per vaccinee was considered an intractable problem for the immune system. Hence, 20 epitopes was the limit of calculation for epitope breadth in all analyses presented here.

It is therefore not surprising that increasing the breadth of T cell recognition might be associated with better control of viremia, since the more epitopes recognized the more difficult it would be for the virus to find multiple escape mutations of relatively low fitness costs. A less obvious and perhaps more biologically cogent reason behind the breadth association with viral control is that certain T cell epitopes may be more effective than others. Prior studies have implicated T cells targeting certain gene products of the virus (e.g. Gag) as being associated with better control of viremia [43,45,48-50,62]. Further studies have refined these analyses to regions of certain gene products and even particular epitopes. The reasons for this phenomenon could include any or all of the time and timing of gene product expression, the HLA background of the infected individual, the availability of generated epitopes for antigen processing and presentation, and the TCR repertoire. Aside from certain epitopes (particularly in the Gag and Nef proteins) presented by alleles frequently associated with control of viremia (HLA-B2705, -B5701/03, -B5801; [49,50,62,63]) there is a paucity of knowledge relating to exactly which epitopes provide the best protection. Without *a priori *knowledge of epitopes that should be targeted by any given individual, the multi-hit models of vaccine assessment presented here provides a potential alternative. While the single-hit model assumes that all epitopes may be equally effective at mediating infected cell recognition, the 2-hit and 3-hit models assume that only one-half and one-third of all possible T cell epitopes respectively are actually effective in mediating T cell recognition of virally infected target cells and inhibiting viral replication. The data presented here show how the consideration of multiple hit models places a greater onus upon any given vaccine product to provide appropriate coverage of all possible incoming strains of HIV-1. Using the natural sequence based vaccine constructs developed in the MHRP it is shown that if used in mono-valent formulations there is sufficient coverage of the homologous subtype, even within the Env protein if sufficient breadth of the immune response (4-6 epitopes) is generated. Furthermore, multi-valent formulation of the products potentially provides ample coverage (> 90% of strains) even for the global HIV-1 group M epidemic for both Gag and Env when considering the single-hit model. The greater diversity within the Env protein compared with Gag is reflected in the generalized requirement for nearly twice the number of epitopes in Env to reach any level of equivalent coverage. However, if a multi-hit model proves to be an accurate description of T cell requirements for an effective vaccine, then the requirements to meet any level of coverage increase dramatically. For mono-valent formulations both the 2- and 3-hit models place such high requirements upon both Gag (8-12 epitopes for 75% coverage using a 2-hit model) and Env (17-19 epitopes for 75% coverage using a 2-hit model), that generating sufficient breadth would be a difficult proposition. Considering a 3-hit model and 90% coverage of the global epidemic the epitope breadth requirements present what is most likely an insurmountable challenge for the immune system (> 20 epitopes required per protein). Multi-valent formulation of the natural sequences does provide increased benefit in terms of generating potentially more cross-reactive cells, especially when considering subtype-specific vaccine coverage. However, as far as global group M coverage is concerned multi-valent formulation and delivery of the natural sequence based products is the most realistic means of ensuring sufficient coverage if multiple exact T cell epitope matches are required for vaccine efficacy.

While quite sobering, these data serve to underscore the practical requirements for a T cell-based vaccine to be efficacious in a broader setting and also provides meaningful tools for assessing those requirements. This kind of analysis could also be directly applied to any kind of computationally derived vaccine insert sequences such as consensus, center-of-tree, mosaic, conserved element or string epitope concatenated sequences [26,29-41]. Such approaches offer a means of maximizing vaccine coverage at the single epitope level and by direct inference would also improve coverage for multiple epitope and multiple-hit requirement models of vaccine coverage. The only caveat that needs to be fulfilled for the computational approaches is proof-of-concept studies in human trials that verify the products can indeed be immunogenic. Epitopes targeting sites of high-depth rather low depth of coverage is embodied in the mosaic, center-of-tree and conserved elements vaccine approaches. Since, the processing of antigens that ultimately results in the presentation of epitopes is not fully understood, a critical issue for the center-of-tree and conserved elements vaccine constructs in particular is a strategy for assembling the peptide fragments for operational vaccine delivery. Simple concatenation of peptide fragments to produce embedded epitopes in the context of the assembled fragments is not sufficient as was demonstrated by the poor immunogenicity observed in two recent trials of such an approach [35,36]. Mosaic sequence based products have at least been shown to be immunogenic for very broad T cell responses in pre-clinical studies in primates [55,56]. The present study has been restricted to the inclusion of natural sequence-based products in the MHRP product development plan (MVA vector-based). These products have all been shown to be immunogenic for T cell responses in pre-clinical studies and in particular, the MVA-CMDR product has been shown to be immunogenic for T cell responses as both a prime and a boost in human phase I clinical trials [31,53,64]. An added rationale for at least maintaining the native Env protein is conformation for antibody production. A critical assumption that the multi-valent analyses have been predicated upon is the notion that each unique variant of an epitope within the cocktail is either immunogenic or cross-reactive with other variants of the same epitope in the cocktail. In cases where this assumption does not hold, the multi-valent analysis presented here may overestimate epitope depth of recognition. However, as discussed previously this possible over-estimation may be counter-acted numerically by the high stringency of exact epitope recognition that the model is based upon. In the setting of a vaccine trial mapping of the response permits analysis not only of the number of T cell epitopes, but also the epitope repertoire and depth of recognition. As shown in this study accurately mapping T cell responses adds the important dimension of depth of epitope recognition to the analysis. Knowledge of at least the approximate minimal epitope (down to 15-mer resolution), allows both a more accurate theoretical estimation of potential depth of recognition and further experimental validation of the cross-reactivity of a response using designed peptide variants.

The T cell epitope breadth analysis presented here also highlights the issue of formulation and delivery of multi-valent vaccine products. While the simplest approach for delivery would be co-formulation for both priming and boosting vaccinations, a multi-valent product may benefit from priming with a single valency product and sequential boosting with the heterologous products. Such an approach might focus the T cell response upon the boosting of cross-reactive T cell responses at the expense of boosting potentially immunodominant responses with limited cross-reactivity. The heterologous prime-boost approach has already been shown to be effective in generating high magnitude T cell responses in several human vaccine trials [64-66]. The recent successful Thai phase III trial (RV144) provides an important example of how the role of T cells and their cross-reactive capacity must be taken into account even in the setting of a heterologous prime-boost approach involving a recombinant protein as one of the formulations [17]. A vaccine that generates antibodies as the likely mediator of its efficacy would still require appropriate T cell help for formation of an adequate antibody repertoire [67-70]. The HIV vaccine field is currently moving forward from the pre-clinical phase into the clinical testing phase of vaccines with increasing complexity in terms of both formulation and the mechanism(s) by which they mediate protective effects. The role of T cells in all of these scenarios must be addressed whether it is indirectly as help for antibody production against multimeric envelope proteins, or directly as effector or effector-memory cells generated by recombinant viral vectors [2,6,51,71,72].

There are several further refinements and caveats that need to be kept in mind when such analysis is performed. An implicitly important variable in this analysis is the query database. This study has made use of the LANL HIV sequence database without bias for the prevalence of any given subtype. However, this database is heavily biased towards subtypes B and C in terms of raw sequence constituency. Hence, more nuanced analyses for vaccines targeted for East Africa, West Africa, Asia and eastern Europe, would benefit from more expansive query datasets that include better sampling of subtypes A, D, G, CRF01_AE and CRF01_AG. This highlights the importance of sequence sampling and the dataset used as the basis for the coverage requirement calculations. Experimentally, there is also a need to discern the relative contributions of CD4^+ ^versus CD8^+ ^T cell responses. CD8^+ ^T cells are generally considered the effector cells responsible for viral control and clearance and therefore the specific contribution of these cells to the overall should be specifically assessed. The most robust and practical assay for epitope enumeration and identification in a vaccine trial is the IFNγ Elispot assay. However, this assay is not readily adaptable to discerning the phenotype of the responding cells without significant sample usage. Therefore, high throughput flow cytometry-based assays that can measure epitope depth and breadth as well as provide high content phenotypic analysis of the responding cell populations are required.

## Conclusions

In summary, we have shown here that appraisal of epitope breadth and depth of recognition should be an important metric that is considered when laboratory assessment of HIV vaccine trials is performed. Furthermore, the incorporation of a multi-hit model into the theoretical breadth analysis helps to set the metrics for broadly specific T cell responses in the absence of mechanistic data that explains the biological underpinnings of why high breadth responses may be advantageous. The suitability for a particular vaccine and multi-valent formulation can be also assessed during the design phase of a clinical trial as well as during the execution phase. Moreover, this study shows that multi-valent formulation, or heterologous prime-boost strategies, may extend the utility of vaccine products based upon natural sequences. While it is clearly shown that mono-valent natural sequence based products face a potentially intractable problem for providing appropriate coverage for the global HIV-1 pandemic, multi-valent formulation can improve the utility of these extant vaccine products, especially in an epidemic consisting of a single subtype. Given that computationally designed vaccine products optimized for maximizing coverage, such as mosaic products, will soon be tested in human clinical trials it remains paramount that robust and practical breadth assessment is included in the testing schema for comparing and contrasting these and other approaches for successful HIV vaccine implementation.

## Competing interests

The authors declare that they have no competing interests.

## Authors' contributions

JRC: Conceived and designed the study, developed the model and wrote the manuscript. MAM: Wrote the manuscript and provided important intellectual input into the overall concept of the study. MLR and NLM: Provided support for the study and participated in interpretation of the data and its implication for study vaccine design and assessment. All authors read and approved the final manuscript.

## Supplementary Material

Additional file 1 **Subtype and isolate sequence of origin of the natural sequence-based HIV vaccine products assessed in the study**. Click here for file

Additional file 2 **Comparison of Epicover algorithm with a "hit/no-hit" model of N-mer epitope identity matching**. A stringent "hit/no-hit" model of 10-mer coverage was applied to a dataset of 2285 Gag sequences using a tetra-valent ACDE natural sequence vaccine formulation as the query set. As shown below a near-Gaussian distribution of the percent coverage of all isolates in the dataset was obtained. Near identical mean coverage results were obtained using the Epicover algorithm (as implemented in the LANL HIV Sequence Database) and a larger dataset of 3585 Gag sequences. While a broad distribution of "hits per isolate" was obtained (range = 13%-75%), the near identical mean coverage results for the Epicover algorithm and the "hit/no-hit" model resulted in no difference between the two methods for the calculation of epitope coverage requirements and the multiple epitope hit requirement model. Click here for file

Additional file 3 **Global group M Gag coverage analysis**. Potential HIV isolate coverage provided by mono-valent (panels **A**, **B **and **C**), di-valent (panels D, E and F) and multi-valent (Panels **G**, **H **and **I**) formulations of the four natural sequence based products is shown. Theoretical coverage (90% in the examples shown here) is profoundly dependent upon the number of epitopes generated and the number of exact epitope (10-mer) matches that are required for infected cell recognition. For example if a 1-Hit model is considered, then the tetra-valent product would reach 90% coverage by generating four epitopes per subject on average (Panel **G**, orange diamonds). In contrast, if a 3-Hit model is considered the mono-valent subtype A product does not reach 90% coverage even if 20 epitopes are generated on average per subject (Panel **C**, blue circles). Click here for file

Additional file 4 **Global group M Env coverage analysis**. Potential HIV isolate coverage provided by mono-valent (Panels **A**, **B **and **C**), di-valent (panels D, E and F) and multi-valent (panels G, H and I) formulations of the four natural sequence based products is shown. Theoretical coverage (90% in the examples shown here) is again dependent upon the number of epitopes generated but there is a much greater epitope requirement than for Gag. For example if a 1-Hit model is considered, then the tetra-valent product would reach 90% coverage by generating 7 epitopes per subject on average (Panel **G**, orange diamonds). If a 3-Hit model is considered the mono-valent subtype products face an intractable problem with extreme epitope requirements for 90% global coverage (Panel **C**). Even the multi-valent products have stringent epitope requirements (17-20) for reaching 90% coverage (Panel **I**). Click here for file

Additional file 5 **Gag epitope mapping for post-*in vitro *stimulated cells from a vaccine trial responder subject**. PBMC from a vaccinee in the RV158 trial (received 3x rMVA-CMDR) were subjected two rounds of *in vitro *stimulation, first with rMVA-CMDR for 14 days, then with autologous, irradiated BLCL pulsed with peptide pools matching the Gag (CM240) insert in the vaccine. Each stimulation cycle was 14 days, with rIL-7 added during the first week and rIL-2 added during the second week. Effector cells were tested for responses to a matrix of peptides (11 × 11 peptide pools) matching the Gag insert sequence. Epitopes were counted and identified from the de-convoluted peptide matrix (Panels **A **and **B**) in an IFN-γ Elispot assay. Two epitopes were identified within the Gag-specific effector cells and are shown in Panel **C**. Click here for file

Additional file 6 **Env epitope mapping for post-*in vitro *stimulated cells from a vaccine trial responder subject**. PBMC from a vaccinee in the RV158 trial (received 3x rMVA-CMDR) were subjected two rounds of *in vitro *stimulation, first with rMVA-CMDR for 14 days, then with autologous, irradiated BLCL pulsed with peptide pools matching the Env (CM235) insert in the vaccine. Each stimulation cycle was 14 days, with rIL-7 added during the first week and rIL-2 added during the second week. Effector cells were tested for responses to a matrix of peptides (14 × 13 peptide pools) matching the Env insert sequence. Epitopes were counted and identified from the de-convoluted peptide matrix (Panels **A **and **B**) in an IFN-g Elispot assay. Two epitopes were identified within the Env-specific effector cells and are shown in Panel **C**. Click here for file
