# Supplementary material for: Defining epitope coverage requirements for T cell-based HIV vaccines: Theoretical considerations and practical applications
Source: J Transl Med. 2011 Dec 8;9:212. doi: 10.1186/1479-5876-9-212 (PMC3284408; doi:10.1186/1479-5876-9-212)
Supplement: Additional file 1 — Subtype and isolate sequence of origin of the natural sequence-based HIV vaccine products assessed in the study. [file 1479-5876-9-212-S1.PDF]

Additional File 1. Subtype and isolate sequence of origin of the natural sequence-based HIV vaccine products assessed in the study.

Product	Subtype of Insert (Country)	Isolate of Insert		GenBank Acc#
MVA-KEA	A	Gag/Pol:	KER2008	AF457052
	(Kenya)	Env:	KNH1144	AF457066
MVA-TZC	C	Gag/Pol:	TZA246	AY253308
	(Tanzania)	Env:	TZA125	AY253304
MVA-UGD	D	Gag/Pol:	AO3349	AF484518
	(Kenya)	Env:	AO7412	AF484477
MVA-CMDR	CRF01_AE	Gag/Pol:	CM240	U54771
	(Thailand)	Env:	CM235	AY736837
