# Supplementary material for: Defining epitope coverage requirements for T cell-based HIV vaccines: Theoretical considerations and practical applications
Source: J Transl Med. 2011 Dec 8;9:212. doi: 10.1186/1479-5876-9-212 (PMC3284408; doi:10.1186/1479-5876-9-212)

Additional File 2: A stringent "hit/no-hit" model of 10-mer coverage was applied to a dataset of 2285 Gag sequences using a tetra-valent ACDE natural sequence vaccine formulation as the query set. As shown below a near-Gaussian distribution of the percent coverage of all isolates in the dataset was obtained. Near identical mean coverage results were obtained using the Epicover algorithm (as implemented in the LANL HIV Sequence Database) and a larger dataset of 3585 Gag sequences. While a broad distribution of "hits per isolate" was obtained (range = 13%-75%), the near identical mean coverage results for the Epicover algorithm and the "hit/no-hit" model resulted in no difference between the two methods for the calculation of epitope coverage requirements and the multiple epitope hit requirement model.

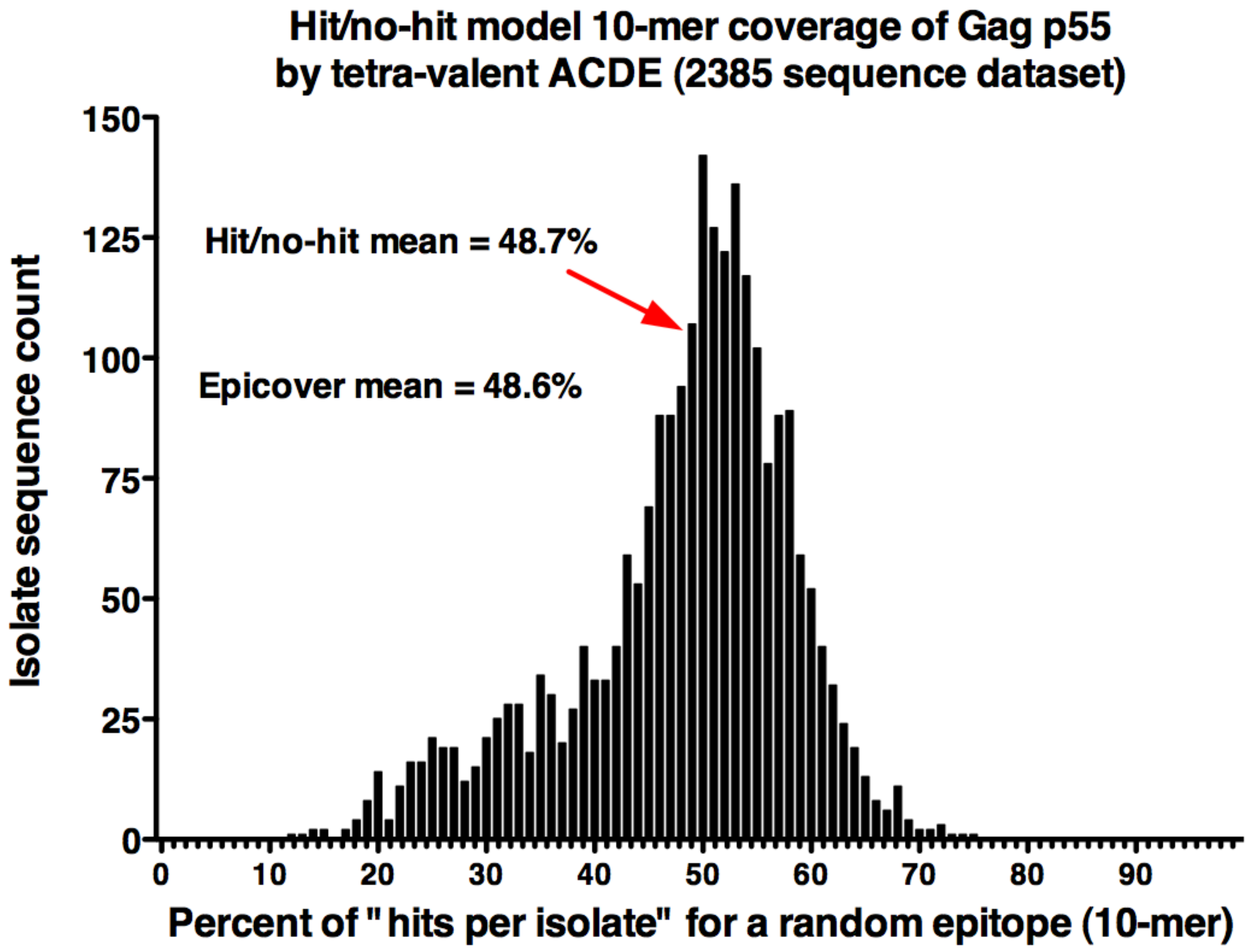

Supplement: Additional file 2 — Comparison of Epicover algorithm with a "hit/no-hit" model of N-mer epitope identity matching. A stringent "hit/no-hit" model of 10-mer coverage was applied to a dataset of 2285 Gag sequences using a tetra-valent ACDE natural sequence vaccine formulation as the query set. As shown below a near-Gaussian distribution of the percent coverage of all isolates in the dataset was obtained. Near identical mean coverage results were obtained using the Epicover algorithm (as implemented in the LANL HIV Sequence Database) and a larger dataset of 3585 Gag sequences. While a broad distribution of "hits per isolate" was obtained (range = 13%-75%), the near identical mean coverage results for the Epicover algorithm and the "hit/no-hit" model resulted in no difference between the two methods for the calculation of epitope coverage requirements and the multiple epitope hit requirement model. [file 1479-5876-9-212-S2.PDF]
