# Supplementary material for: Defining epitope coverage requirements for T cell-based HIV vaccines: Theoretical considerations and practical applications
Source: J Transl Med. 2011 Dec 8;9:212. doi: 10.1186/1479-5876-9-212 (PMC3284408; doi:10.1186/1479-5876-9-212)

Additional File 3: Global group M Gag coverage analysis. Potential HIV isolate coverage provided by mono-valent (panels A, B and C), di-valent (panels D, E and F) and multi-valent (panels G, H and I) formulations of the four natural sequence based products is shown. Theoretical coverage (90% in the examples shown here) is profoundly dependent upon the number of epitopes generated and the number of exact epitope (10-mer) matches that are required for infected cell recognition. For example if a 1-Hit model is considered, then the tetra-valent product would reach 90% coverage by generating four epitopes per subject on average (panel G, orange diamonds). In contrast, if a 3-Hit model is considered the mono-valent subtype A product does not reach 90% coverage even if 20 epitopes are generated on average per subject (panel C, blue circles).

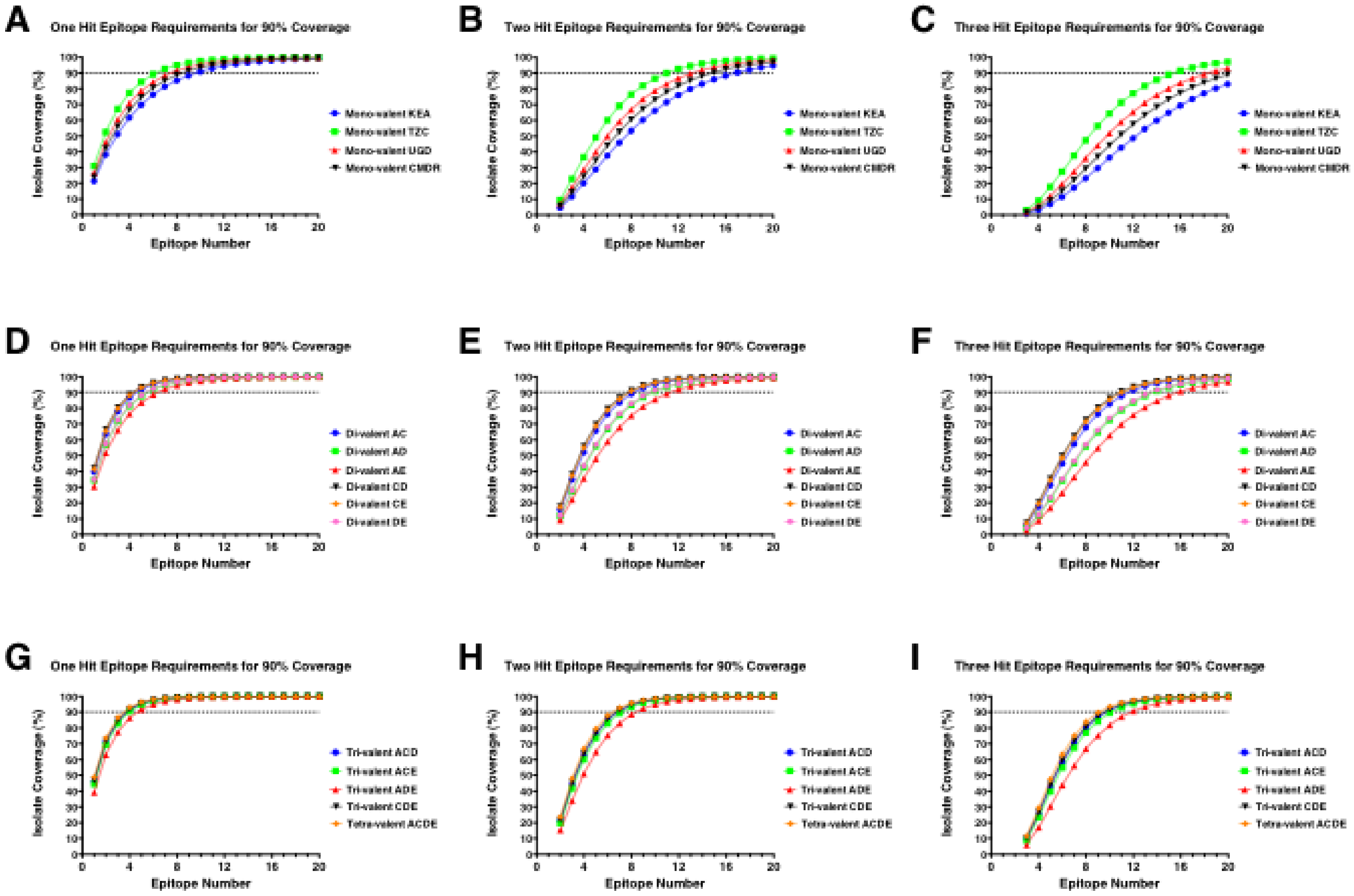

Supplement: Additional file 3 — Global group M Gag coverage analysis. Potential HIV isolate coverage provided by mono-valent (panels A, B and C), di-valent (panels D, E and F) and multi-valent (Panels G, H and I) formulations of the four natural sequence based products is shown. Theoretical coverage (90% in the examples shown here) is profoundly dependent upon the number of epitopes generated and the number of exact epitope (10-mer) matches that are required for infected cell recognition. For example if a 1-Hit model is considered, then the tetra-valent product would reach 90% coverage by generating four epitopes per subject on average (Panel G, orange diamonds). In contrast, if a 3-Hit model is considered the mono-valent subtype A product does not reach 90% coverage even if 20 epitopes are generated on average per subject (Panel C, blue circles). [file 1479-5876-9-212-S3.PDF]
