# Supplementary material for: Defining epitope coverage requirements for T cell-based HIV vaccines: Theoretical considerations and practical applications
Source: J Transl Med. 2011 Dec 8;9:212. doi: 10.1186/1479-5876-9-212 (PMC3284408; doi:10.1186/1479-5876-9-212)

Additional File 4: Global group M Env coverage analysis. Potential HIV isolate coverage provided by mono-valent (panels A, B and C), di-valent (panels D, E and F) and multi-valent (panels G, H and I) formulations of the four natural sequence based products is shown. Theoretical coverage (90% in the examples shown here) is again dependent upon the number of epitopes generated but there is a much greater epitope requirement than for Gag. For example if a 1-Hit model is considered, then the tetra-valent product would reach 90% coverage by generating 7 epitopes per subject on average (panel G, orange diamonds). If a 3-Hit model is considered the mono-valent subtype products face an intractable problem with extreme epitope requirements for 90% global coverage (panel C). Even the multi-valent products have stringent epitope requirements (17-20) for reaching 90% coverage (panel I).

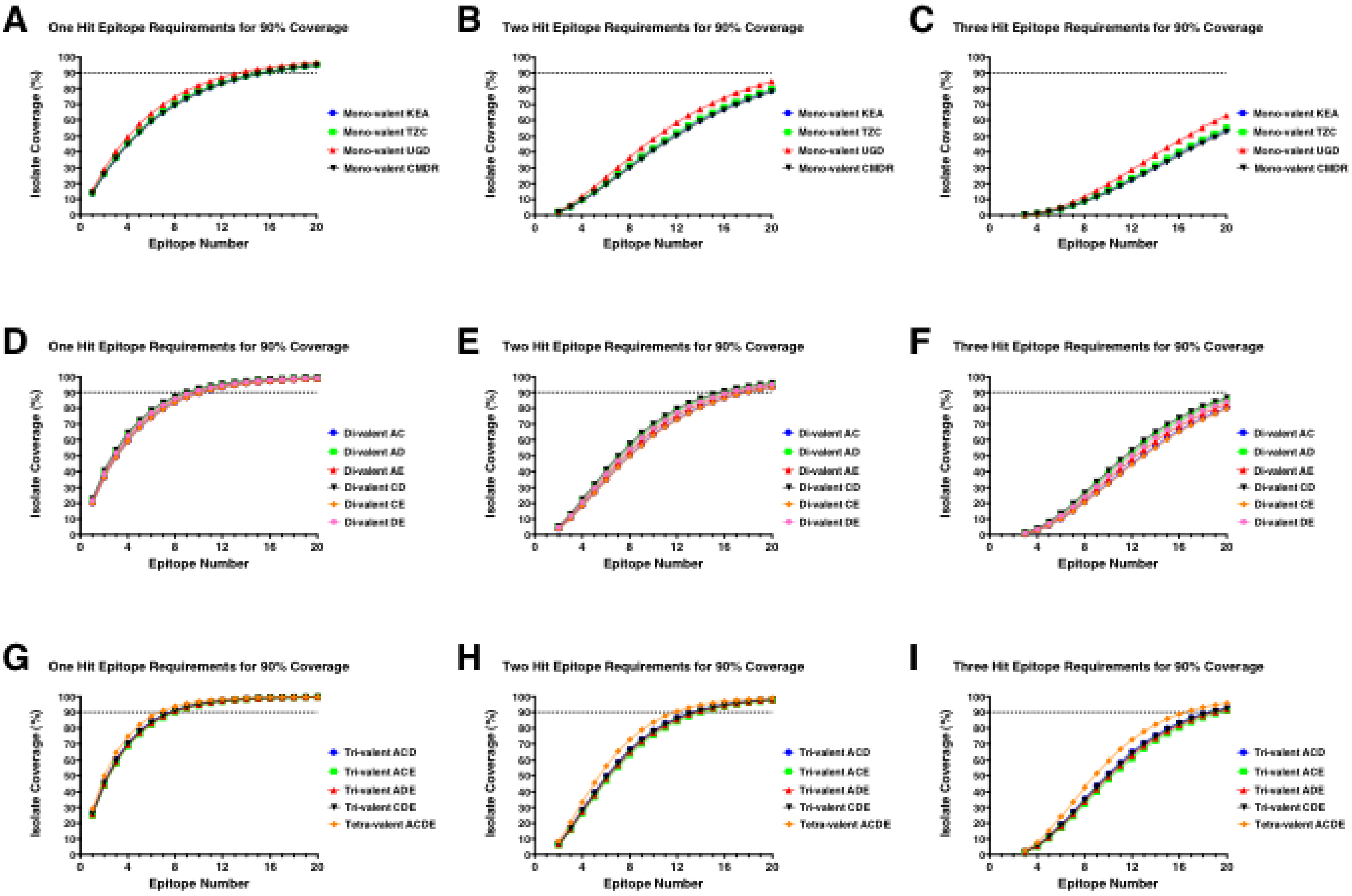

Supplement: Additional file 4 — Global group M Env coverage analysis. Potential HIV isolate coverage provided by mono-valent (Panels A, B and C), di-valent (panels D, E and F) and multi-valent (panels G, H and I) formulations of the four natural sequence based products is shown. Theoretical coverage (90% in the examples shown here) is again dependent upon the number of epitopes generated but there is a much greater epitope requirement than for Gag. For example if a 1-Hit model is considered, then the tetra-valent product would reach 90% coverage by generating 7 epitopes per subject on average (Panel G, orange diamonds). If a 3-Hit model is considered the mono-valent subtype products face an intractable problem with extreme epitope requirements for 90% global coverage (Panel C). Even the multi-valent products have stringent epitope requirements (17-20) for reaching 90% coverage (Panel I). [file 1479-5876-9-212-S4.PDF]
