# Supplementary material for: Defining epitope coverage requirements for T cell-based HIV vaccines: Theoretical considerations and practical applications
Source: J Transl Med. 2011 Dec 8;9:212. doi: 10.1186/1479-5876-9-212 (PMC3284408; doi:10.1186/1479-5876-9-212)
Supplement: Additional file 5 — Gag epitope mapping for post-in vitro stimulated cells from a vaccine trial responder subject. PBMC from a vaccinee in the RV158 trial (received 3x rMVA-CMDR) were subjected two rounds of in vitro stimulation, first with rMVA-CMDR for 14 days, then with autologous, irradiated BLCL pulsed with peptide pools matching the Gag (CM240) insert in the vaccine. Each stimulation cycle was 14 days, with rIL-7 added during the first week and rIL-2 added during the second week. Effector cells were tested for responses to a matrix of peptides (11 × 11 peptide pools) matching the Gag insert sequence. Epitopes were counted and identified from the de-convoluted peptide matrix (Panels A and B) in an IFN-γ Elispot assay. Two epitopes were identified within the Gag-specific effector cells and are shown in Panel C. [file 1479-5876-9-212-S5.PDF]

Additional File 5: Gag epitope mapping for post-*in vitro* stimulated cells from a vaccine trial responder subject. PBMC from a vaccinee in the RV158 trial (received 3x rMVA-CMDR) were subjected two rounds of *in vitro* stimulation, first with rMVA-CMDR for 14 days, then with autologous, irradiated BLCL pulsed with peptide pools matching the Gag (CM240) insert in the vaccine. Each stimulation cycle was 14 days, with rIL-7 added during the first week and rIL-2 added during the second week. Effector cells were tested for responses to a matrix of peptides (11 x 11 peptide pools) matching the Gag insert sequence. Epitopes were counted and identified from the de-convoluted peptide matrix (panels A and B) in an IFN-γ Elispot assay. Two epitopes were identified within the Gag-specific effector cells and are shown in panel C.

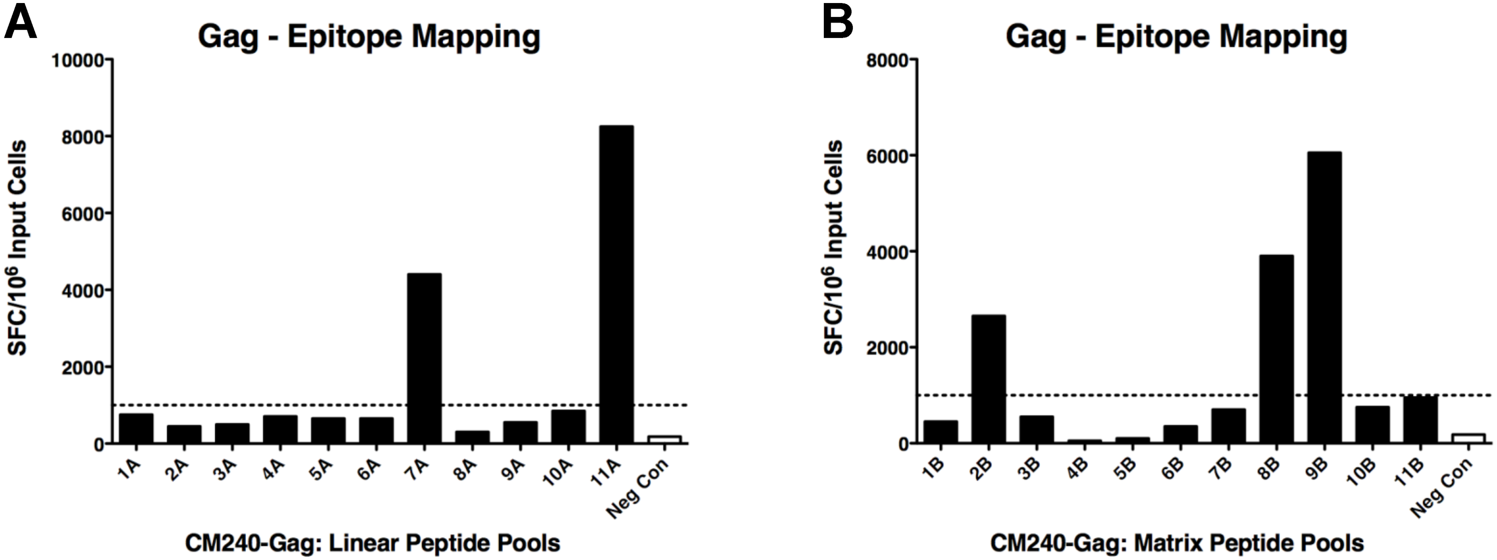

C Mapped and identified Gag epitopes. Sequences of the mapped Gag epitopes are shown together with the percent 10-mer identity each epitope shares with the denoted number of intra-subtype (CR01_AE) and global group M sequences.

Epitope	Sequence	Intra-subtype Gag Coverage 87 Sequences	Global Gag Coverage 2861 Sequences
1	VDRFYKTLRAE	89.7%	42.1%
2	NFPQSRPEPTAPPAE	58.6%	42.4%
