# Supplementary material for: Defining epitope coverage requirements for T cell-based HIV vaccines: Theoretical considerations and practical applications
Source: J Transl Med. 2011 Dec 8;9:212. doi: 10.1186/1479-5876-9-212 (PMC3284408; doi:10.1186/1479-5876-9-212)
Supplement: Additional file 6 — Env epitope mapping for post-in vitro stimulated cells from a vaccine trial responder subject. PBMC from a vaccinee in the RV158 trial (received 3x rMVA-CMDR) were subjected two rounds of in vitro stimulation, first with rMVA-CMDR for 14 days, then with autologous, irradiated BLCL pulsed with peptide pools matching the Env (CM235) insert in the vaccine. Each stimulation cycle was 14 days, with rIL-7 added during the first week and rIL-2 added during the second week. Effector cells were tested for responses to a matrix of peptides (14 × 13 peptide pools) matching the Env insert sequence. Epitopes were counted and identified from the de-convoluted peptide matrix (Panels A and B) in an IFN-g Elispot assay. Two epitopes were identified within the Env-specific effector cells and are shown in Panel C. [file 1479-5876-9-212-S6.PDF]

Additional File 6: Env epitope mapping for post-*in vitro* stimulated cells from a vaccine trial responder subject. PBMC from a vaccinee in the RV158 trial (received 3x rMVA-CMDR) were subjected two rounds of *in vitro* stimulation, first with rMVA-CMDR for 14 days, then with autologous, irradiated BLCL pulsed with peptide pools matching the Env (CM235) insert in the vaccine. Each stimulation cycle was 14 days, with rIL-7 added during the first week and rIL-2 added during the second week. Effector cells were tested for responses to a matrix of peptides (14 x 13 peptide pools) matching the Env insert sequence. Epitopes were counted and identified from the de-convoluted peptide matrix (panels A and B) in an IFN-γ Elispot assay. Two epitopes were identified within the Env-specific effector cells and are shown in panel C.

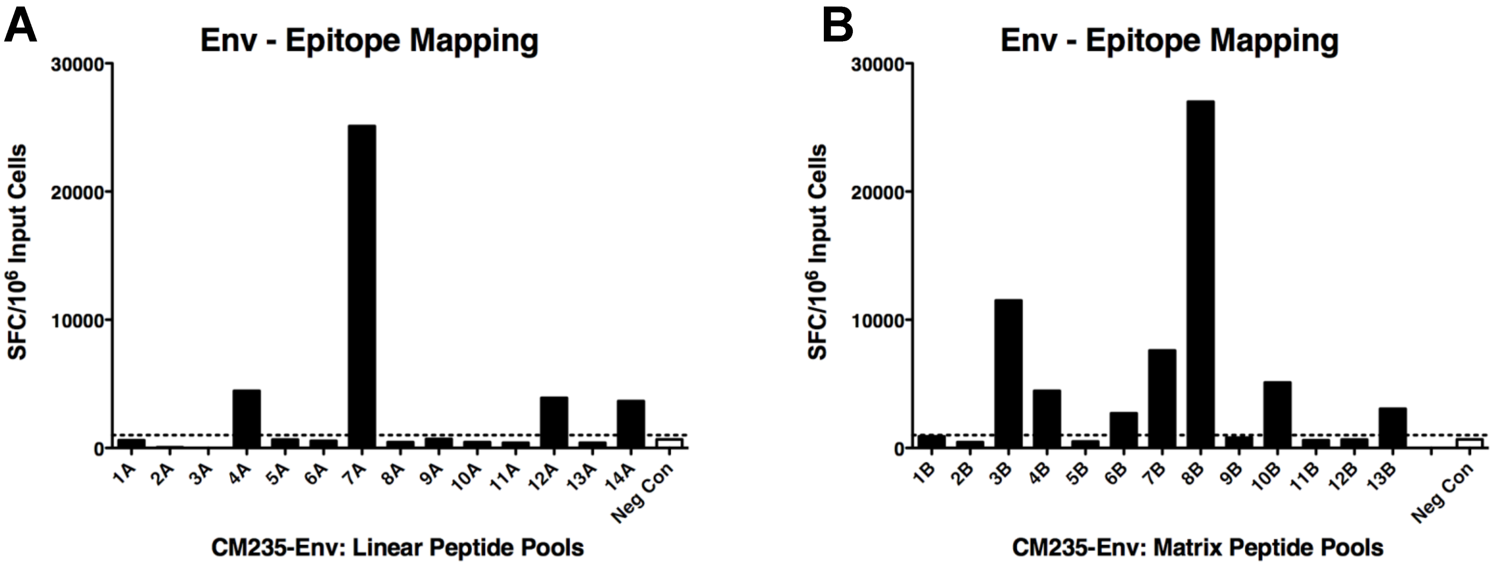

C

Mapped and identified Env epitopes. Sequences of the mapped Env epitopes are shown together with the percent 10-mer identity each epitope shares with the denoted number of intra-subtype (CR01_AE) and global group M sequences.

Epitope	Sequence	Intra-subtype Env Coverage 111 Sequences	Global Env Coverage 2230 Sequences
1	VHALFYKLDIVPIED	20.70%	6.9%
2	EKLKEHFNNKTIIFQ	0%	0%
3	EISNYTNQIYEILTE	45.10%	2.8%
4	EPDRSERIEEGGGEQ	30.6%	5.7%
